# Effect of Alpha-Hederin, the active constituent of *Nigella sativa*, on miRNA-126, IL-13 mRNA levels and inflammation of lungs in ovalbumin-sensitized male rats

**Published:** 2016

**Authors:** Maryam Fallahi, Rana Keyhanmanesh, Amir Mahdi Khamaneh, Mohammad Ali Ebrahimi Saadatlou, Saeideh Saadat, Hadi Ebrahimi

**Affiliations:** 1 *Department of Physiology, Faculty of Medicine, Tabriz University of Medical Sciences, Tabriz, Iran*; 2 *Medical Education Research Center, Tabriz University of Medical Sciences, Tabriz, Iran*; 3 *Department of molecular medicine, School of advanced medical sciences, Tabriz University of Medical Sciences, Tabriz, Iran*; 4 *Department of basic sciences, College of veterinary medicine, Tabriz branch, Islamic Azad University, Tabriz, Iran*; 5 *Department of Physiology, Faculty of Medicine, Zahedan University of Medical Sciences, Zahedan, Iran*; 6 *Tuberculosis and lung diseases research center, Tabriz University of Medical Sciences, Tabriz, Iran*

**Keywords:** *Alpha-hederin*, *MiRNA-126*, *IL-13 mRNA*, *Ovalbumin*, *Thymoquinone*, *Asthma*

## Abstract

**Objective:**

In previous studies the therapeutic effects of *Nigella sativa* have been demonstrated on asthmatic animals. In the present study, the preventive effect of single dose of alpha-hederin, its active constituent, has been evaluated on lung inflammation and some inflammatory mediators in lungs of ovalbumin sensitized rat in order to elicit its mechanism.

**Materials and Methods:**

Forty rats were randomly grouped in 4 groups; control (C), sensitized (S), sensitized pretreated groups with thymoquinone (3 mg/kg i.p., S+TQ) and alpha-hederin (0.02 mg/kg i.p., S+AH). Levels of IL-13 mRNA and miRNA-126 in lung tissue and its pathological changes in each group were assessed.

**Results:**

Elevated levels of miRNA-126, IL-13 mRNA and pathological changes were observed in the sensitized group compared to the control group (p<0.001 to p<0.05). All of these factors were significantly reduced in S+TQ and S+AH groups in comparison to S group (p<0.001 to p<0.05). Although alpha-hederin decreased the levels of miRNA-126, IL-13 mRNA and pathological changes in comparison with thymoquinone, the results were statistically not significant.

**Conclusion:**

The results suggested that alpha-hederin had preventive effect on sensitized rats like thymoquinone. It may intervene in miRNA-126 expression, which consequently could interfere with IL-13 secretion pathway leading to a reduction in inflammatory responses.

## Introduction

Asthma is an airways chronic inflammatory disease which has recently become epidemic in developed countries (Mattes et al., 2009 ▶). Both exposure to environmental triggers and genetic factors can attribute to asthmatic pathogenesis (Oglesby et al., 2010 ▶). The symptoms include wheezing and breathlessness along with enhanced airway hyper-responsiveness caused by a vast range of stimuli. The potential mechanism causing asthma is that the innate immune system senses foreign and dangerous allergens and responds with allergen-specific CD4+ T helper-2 lymphocyte (Mattes et al., 2009 ▶). The inflammation can be caused by mast cells and eosinophils infiltrated, an exaggerated T helper-2 (Th2) response and the actions performed by their secreted cytokines. The Th2 cytokines linked to mucus hyper secretion, eosinophil infiltration, hyper-responsiveness and elevated levels of IgE, are IL-4, IL-5, IL-9, and IL-13 (Oglesbyet al., 2010 ▶;Rebane et al., 2013). As reduction or elimination of Th2 functions can reduce allergic inflammatory responses in the early stage of asthma (Bosnjak et al., 2011 ▶), these factors can be useful targets to manage the severity of the disease (Barnes, 2001 ▶).

MicroRNAs (miRNAs), small non-coding RNA molecules, can negatively regulate the expression of different genes (Angulo et al., 2012 ▶). Many studies demonstrated that miRNAs can modulate essential physiological processes leading to expression or suppression of a cascade of molecules (Yang, 2008 ▶). The role of miRNAs in mediating different stages of various types of disease ranging from malignant neoplasms to cardiovascular disorders has been of interest throughout the years (Collisonet al., 2011 ▶). As miRNAs participate in pathogenesis of pulmonary disease such as pulmonary fibrosis, lung cancer, chronic obstructive pulmonary disease and asthma, further studies on the role of miRNAs can give us a perspective into new therapeutic and diagnostic tools (Anguloet al., 2012 ▶). MiRNA-126 is amongst the ones being studied according to asthma (Collisonet al., 2011b ▶). Previous study showed that asthmatic inflammation was associated with upregulation of miRNA-126 (Kabesch et al., 2012).

The common anti-inflammatory drugs are associated with side effects and less potency in asthma treatment. Therefore herbal medicine therapy has recently become the center of attention (Gepdiremen et al., 2004 ▶). One of these medicinal herbs with anti-asthmatic effect is *Nigella sativa* and its bioactive constituent, alpha-hederin (Rooney and Ryan, 2005 ▶). Alpha-hederin is also one of the principal bioactive constituents of Hedera helix. This plant, known as English ivy is a member of Araliaceae family and possesses therapeutic effects such as antihelmintic, antifungal, leishmanicidic antibacterial, spasmolytic, bronchodilating, acute and chronic anti-inflammatory effects (Gepdiremen et al., 2004 ▶). These effects mostly rise from triterpene saponins existing in this plant (Hocaoglu et al., 2012 ▶). Alpha-hederin is one of the saponins derived from Hedera Helix which has recently been identified as the main constituent (Wolf et al., 2011 ▶).

As IL-13 is central to the progression of asthma and many miRNAs such as miRNA-126 can regulate its production directly and indirectly (Greenet al., 2013 ▶), in this study we have evaluated the preventive effect of alpha -Hederin on lung pathology, IL-13 mRNA and miRNA-126 in ovalbumin-sensitized male rats in order to elicit its mechanism.

## Materials and Methods

**Experimental design**

40 male Wistar rats weighing 200-250 grams were used in the present study. The animals were held in PVC cages following 12:12 light/dark cycle at 20± 2 C with free access to water and rodent chow. After 10 days of acclimatization, animals were randomly divided into 4 groups each containing 10 rats; control (C), sensitized (S), sensitized pretreated with thymoquinone (Sigma Chemical Ltd., UK; S+TQ) and alpha- hederin (Extrasynthese Co., France; S+AH).

The rats were sensitized to Ovalbumin (OA) in the following manner; on the first day 1 mg OA, dissolved in 1 ml saline, was injected intraperitoneally (i.p). The second injection was administered a week later with the same dose. Starting from day 14, the rats were exposed to a 4% aerosol of OA for 18±1 days, 5 min per day. The aerosol cylinder measured 16 cm high, 19 cm diameter and a volume of 3 liters. The control animals were treated equally with saline instead of OA (Rad et al., 2012 ▶). In pretreated groups, thymoquinone (3 mg/kg) (Keyhanmanesh et al., 2014a ▶) and alpha-hederin (0.02 mg/kg) (Gepdiremen et al., 2005 ▶) were injected intraperitoneally on the 10th day of sensitization. The study was in line with the ethical committee of the Tabriz University of Medical Sciences.

**Total RNA extraction and real time PCR**

One day after the end of sensitization, animals were sacrificed by intraperitoneal injection of ketamine (50 mg/kg) and xylazine (5mg/kg). Then the left lung of each rat was removed, washed with salin and immediately was frozen and floated in liquid nitrogen. After that, tissue homogenization was performed by mortar and pestle with liquid nitrogen transferring. Then total RNA was extracted from lung homogenate by means of TRIzol (Life technologies Corporation, India) and phase separation method. The purity of RNA was measured using a Nanodrop 1000 spectrophotometer (Thermo scientific, Wilmington, DE 19810 USA). Finally Revert Aid First Strand cDNA Synthesis Kit (FermentasGmBH, Leon-Rot, Germany) using random hexamer primers were used to determine IL-13 mRNA expression level.

The expression profile of miR-126 was performed on total RNA extracts by the aid of universal cDNA synthesis kit according to instruction manual of Exiqon. Briefly, total RNA containing microRNA was polyadenylated at first. Then cDNA was synthesized using a poly (T) primer with a 3 degenerate anchor and a 5 universal tag (Exiqon, Vedbaek, Denmark). The cDNA template was then amplified using miR-126-specific and LNA™-enhanced forward and reverse primers. 

Using SYBR Green master mix (Exiqon, Vedbaek, Denmark), each cDNA was used as a template for separate assay for microRNA and mRNA quantitative real-time PCR.

Real-time PCR reactions were performed on a Bio-Rad iQ5 detection System (Bio-Rad, Richmond, CA, USA). Housekeeping beta-glucuronidase gene was used to normalize the amount of PCR products for mRNA samples, and rno-miR-191 for miRNA-126. The 2^-(Ct)^ method was utilized in order to determine relative-quantitative levels of individual mRNAs and miR-126. The results were reported as the fold-difference to the relevant controls (Alipour et al., 2013 ▶).

**Pathological evaluation**

After sacrificing the rats by ketamine/xylazine injection, their right lungs were removed and placed into 10% buffered formalin (37%, Merck, Germany). Seven days later, using an Autotechnicon apparatus, the tissues were dried by passage through 70% ethanol and cleared by xylol. After that the tissues were paraffin blocked. Then the specimens were stained with hematoxylin and eosin (H&E) staining after being cut into 4-μm slices. Finally the tissues were examined under a light microscope (Keyhanmanesh et al., 2010 ▶). 

The pathologic changes in the lung of sensitized and pretreated groups included pneumocyte and fibroblastic hypertrophy and hyperplasia, edematous and degenerative changes, necrosis and airway epithelial denudation, atelectasis, hyperemia, hemorrhage and exudative changes. The pathological changes were scored in the following manner; 0 = no lesion, 1 = slight lesion, 2 = mild lesion, 3 = moderate lesion, 4 = severe lesion.

**Statistical analysis**

All the results were considered as mean ± SEM. The data of four sensitized groups were compared with controls using one-way analysis of variance (ANOVA) with Tukey-Kramer post-test. Furthermore, the data of two pretreated groups were compared with sensitized guinea pigs using one-way analysis of variance (ANOVA) with Tukey-Kramer. The data of alpha-hederin pretreated groups were compared with S+TQ group using the unpaired t-test.

## Results

**Total RNA extraction and real time PCR**

**Figure 1:**
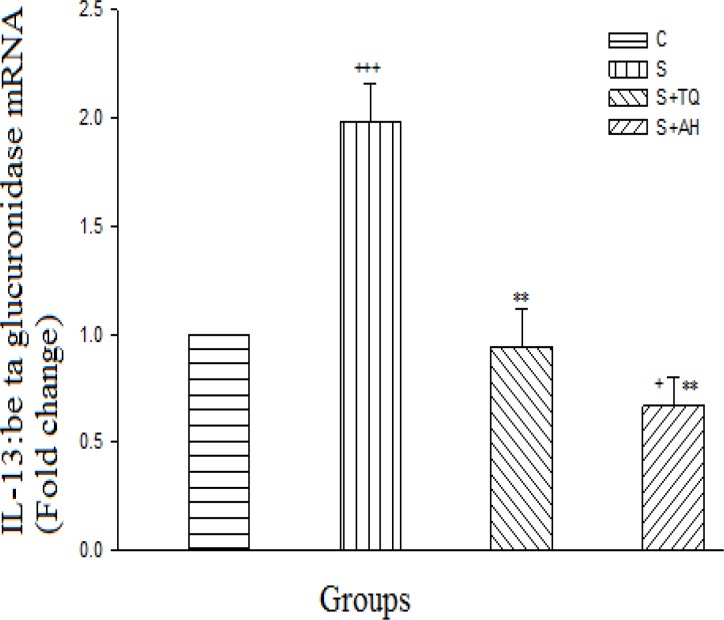
Real-time quantitative RT-PCR analysis of IL-13 mRNA expression level in lung of control (C), sensitized (S), S pretreated with thymoquinone (S+TQ), S pretreated with alpha-hederin (S+AH) rats (for each group, n = 6). Bars represent the mean ± SEM. Statistical differences between different groups *vs.* control: +; p<0.05, +++; p< 0.001. Statistical differences between pretreated groups *vs.* sensitized group: **; p< 0.01

The sensitized group illustrated an extremely significant increase in IL-13 mRNA compared to the control group (p<0.001), while the pretreated group with alpha-hederin had significant decrease (p<0.05). There were significant decreases in both pretreated groups in comparison to the S group (p<0.01). Although alpha-hederin decreased the expression of IL-13 mRNA in comparison with S+TQ group, the result was not statistically significant (Figure 1). 

**Figure 2:**
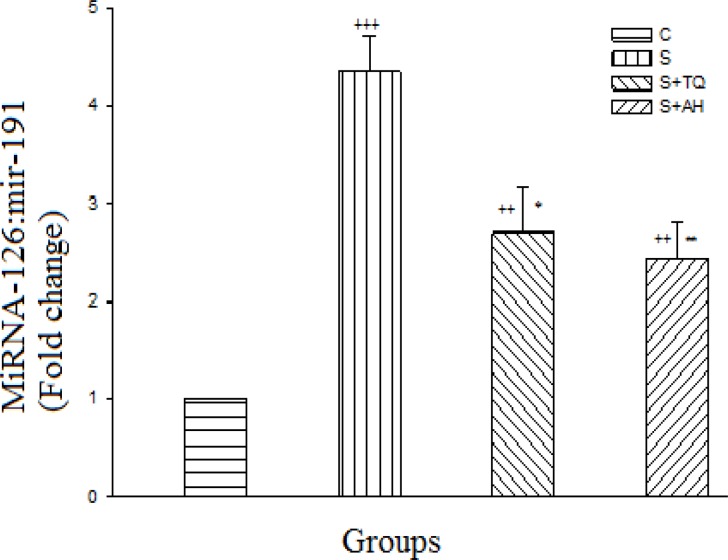
Real-time quantitative RT-PCR analysis of miR-126 expression level in lung of control (C), sensitized (S), S pretreated with thymoquinone (S+TQ), S pretreated with alpha-hederin (S+AH) rats (for each group, n = 6). Bars represent the mean ± SEM. Statistical differences between different groups *vs.* control: ++; p<0.01, +++; p< 0.001. Statistical differences between pretreated groups *vs. *sensitized group: *; p< 0.05, **; p< 0.01.

MiRNA-126 gene expression was increased significantly in all sensitized groups in comparison to C group (p<0.001 to p<0.01). There was significant decrease in pretreated groups compared to sensitized group (p<0.05 for S+TQ group and p<0.01 for S+AH group) however, there were no significant differences between S+AH and S+TQ groups (Figure 2).

**Pathological results**

**Figure 3:**
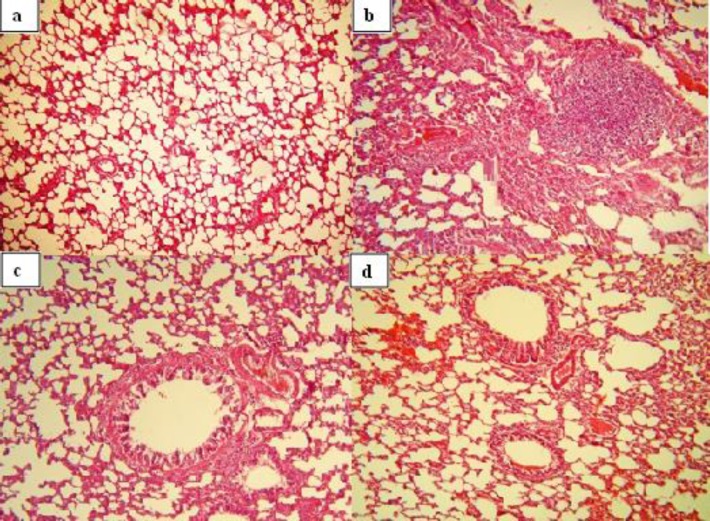
Photographs of a lung specimens in different groups. Normal lung tissue in control group (a, ×120), pneumocyte and fibroblastic hypertrophy and hyperplasia, edematous changes, necrosis and airway epithelial denudation in sensitized group (b, ×120), decreased pneumocyte and fibroblastic hypertrophy and hyperplasia, edematous changes and hyperemia in S+TQ group (c, ×120), decreased pneumocyte and fibroblastic hypertrophy and hyperplasia, hyperemia, hemorrhage, edematous and exudative changes in S+AH (d, ×120

All pathological changes in S group were significantly higher than control (p<0.05 for all, Figure 3). 

**Figure 4:**
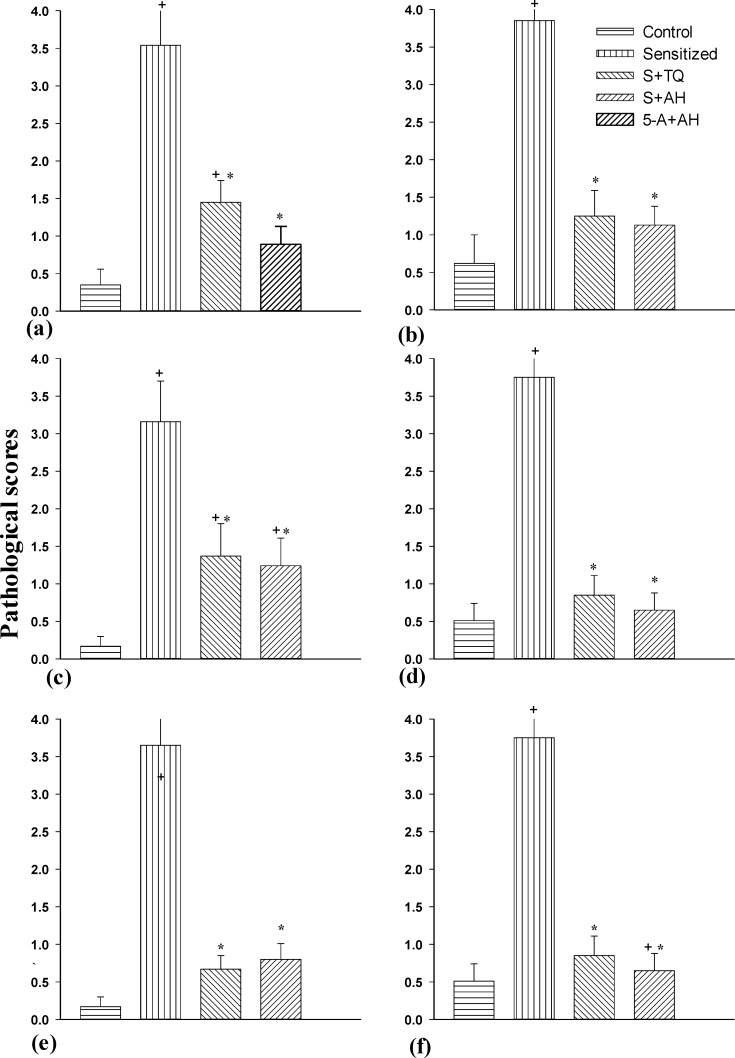
The pneumocyte and fibroblastic hypertrophy and hyperplasia (a), edematous (b) and degenerative (c) changes, necrosis and airway epithelial denudation (d), atelectasis (e), hyperemia, hemorrhage and exudative changes (f) of lungs in control (C), sensitized (S), S pretreated with thymoquinone (S+TQ), S pretreated with alpha-hederin (S+AH) rats (for each group, n = 8). The pathological changes were scored in the following manner; 0 = no lesion, 1 = slight lesion, 2 = mild lesion, 3 = moderate lesion, 4 = severe lesion.

In S+TQ group, pneumocyte and fibroblastic hypertrophy and hyperplasia and degenerative changes were significantly higher than control group (p<0.05). In addition, the degenerative changes, hyperemia, hemorrhage and exudative changes in S+AH group were significantly higher than C group (p<0.05). All of the pathological changes in pretreated groups were significantly lower than the S group (p<0.05). The pneumocyte and fibroblastic hypertrophy and hyperplasia in S+AH were significantly lower than S+TQ group (p<0.05, Figure 4).

## Discussion

The lung IL-13 mRNA level raised in ovalbumin sensitized rats in this investigation. However the underlying mechanisms that cause asthma are not exactly established, T cells, especially Th2 cells, have been proposed to contribute to the induction and maintenance of the inflammatory response. Their activation causes cytokines' release. IL-13 was secreted from these activated cells and improves airway obstruction and develops many of the asthmatic features including airways hyper-responsiveness, mucus hypersecretion, airway eosinophilia and B-cell activation (Tsitsionetal., 2012 ▶). Studies suggest that blockade of IL-13 can normalize the activity of innate immune cells including epithelial cells, dendritic cells and the cells which present allergens to T cells, resulting in a reduction of Th2 immune responses (May et al., 2011 ▶). So this elevated level of IL-13 mRNA was in accordance with these previous studies. The current results showed that administration of thymoquinone and alpha-hederin could prevent this change and confirmed their healing effects. 

Lung miRNA-126 was significantly increased during sensitization protocol of this study. The level of miRNA-126 was very high in patients with asthma following Th2 activity. Its inhibition could prevent the development of airway hypersensitivity by repressing the activation of Th2 (Anguloet al., 2012 ▶) and mucus hypersecretion (Collisonet al., 2011 ▶). Thymoquinone and alpha-hederin in administered dose resulted in the decline of miRNA-126 expression in lungs of sensitized pretreated groups in this investigation. It has been presumed that β2-adrenergic responsiveness raised by alpha-hederin. It could elevate cAMP levels and increase protein kinase A (PKA) which then phosphorylated myosin light-chain kinase (MLCK). Subsequently MLCK sensitivity to calcium would diminish. Moreover, calcium ion release from intracellular stores would be inhibited by cAMP simultaneously reducing calcium entry into cells (Wolf et al., 2011 ▶). 

All pathological changes in the sensitized animals were significantly higher than the control group, which is in line with our previous studies (Keyhanmanesh et al., 2010 ▶). In this study, administeration of thymoquinone and alpha-hederin could ameliorate all pathological features including pneumocytic and fibroblastic hypertrophy and hyperplasia, edematous changes, degenerative changes, necrosis and airway epithelial denudation, atelectasis, hyperemia and hemorrhage and oxidative changes compared to sensitized animals. Many previous studies demonstrated the anti-inflammatory effect of thymoquinone in asthma disease (AbdEl Aziz et al., 2011 ▶; Al-Jawad et al., 2012 ▶; Boskabady et al., 201 ▶0; Keyhanmaneshet al., 2010 ▶ and 2014a ▶-b ▶; Hosseinzadehet al., 2008 ▶; Boskabady and Aslani, 2005 ▶). In addition, Hocaoglu's study had indicated that Hedera Helix could reduce the number of goblet cells and thickness of airway epithelium (Hocaoglu et al., 2012 ▶). Moreover, previous study showed that the injection of two different doses of alpha-hederin could reduce the tracheal responsiveness to methacholine, histamine, and ovalbumin as well as decreasing the total WBC count, eosinophil and basophil numbers, and increasing the number of neutrophil, monocyte and lymphocyte in lung lavage fluid compared to sensitized guinea pigs (Saadat et al., 2015 ▶).

Bars represent the mean ± SEM. Statistical differences between different groups *vs.* control: +; p< 0.05.

Statistical differences between pretreated groups *vs.* sensitized group: *; p< 0.05.

Kumar and his colleagues in previous study demonstrated that antimir-126 could decrease levels of Th2 cytokines such as IL-5 and IL-13 (Kumar et al., 2011 ▶). The decreased level of IL-13 resolved airway inflammation, airway hyper-responsiveness and lowered the mucus metaplasia and subepithelial fibrosis (Green et al., 2013 ▶) and goblet cell hyperplasia (Grünig et al., 2012 ▶). Therefore, it was suggested that alpha-hederin and thymoquinone with used concentrations in current study might decrease miRNA-126 and consequently attenuate the IL-13 secretion pathway leading to decreased inflammatory pathological changes in lung tissue.

There were no significant differences between improvements of lung pathology, miRNA-126 and IL-13 mRNA level caused by alpha-hederin compared to thymoquinone. Also Saadat and her colleagues demonstrated that alpha-hederin had anti-inflammatory and bronchodilatory effects like thymoquinone (Saadat et al., 2015 ▶).

## Conclusion

In conclusion, these data indicated the similar therapeutic effects of alpha-hederin, in used dose, with thymoquinone in sensitized rats. Alpha-hederin may intervene in miRNA-126 expression, which could consequently interfere with IL-13 secretion pathway leading to a reduction in inflammatory responses.
